# Sema4A is implicated in the acceleration of Th17 cell-mediated neuroinflammation in the effector phase

**DOI:** 10.1186/s12974-020-01757-w

**Published:** 2020-03-13

**Authors:** Toru Koda, Akiko Namba, Makoto Kinoshita, Yuji Nakatsuji, Tomoyuki Sugimoto, Kaori Sakakibara, Satoru Tada, Mikito Shimizu, Kazuya Yamashita, Kazushiro Takata, Teruyuki Ishikura, Syo Murata, Shohei Beppu, Atsushi Kumanogoh, Hideki Mochizuki, Tatsusada Okuno

**Affiliations:** 1 Department of Neurology, Osaka University Graduate School of Medicine, 2-2 Yamadaoka, Suita, Osaka 565-0871 Japan; 2 Department of Neurology, Toyama University Hospital, Toyama, Japan; 3 Graduate School of Data Scinece, Shiga University, Shiga, Japan; 4 Department of Neurology, Hoshigaoka Medical Center, Hirakata, Osaka Japan; 5 Department of Respiratory Medicine, Allergy and Rheumatic Diseases, Osaka University Graduate School of Medicine, Suita, Osaka Japan

**Keywords:** Multiple sclerosis, Sema4A, Th17, Experimental autoimmune encephalomyelitis

## Abstract

**Background:**

Sema4A is a regulator of helper T cell (Th) activation and differentiation in the priming phase, which plays an important role in the pathogenesis of experimental autoimmune encephalomyelitis (EAE) and multiple sclerosis (MS). However, the role of Sema4A in the effector phase remains elusive. We aimed to investigate the role of Sema4A at the effector phase in adoptively transferred EAE model. Clinical features and cytokine profiles of MS patients with high Sema4A levels were also examined in detail to clarify the correlation between Sema4A levels and disease activity of patients with MS.

**Methods:**

We adoptively transferred encephalitogenic Th1 or Th17 cells to wild type (WT) or Sema4A-deficient (Sema4A KO) mice and assessed severity of symptoms and cellular infiltration within the central nervous system (CNS). In addition, we analyzed clinical and radiological features (*n* = 201), levels of serum IFN-γ and IL-17A (*n* = 86), complete remission ratio by IFN-β (*n* = 38) in all of relapsing-remitting multiple sclerosis (RRMS) patients enrolled in this study.

**Results:**

Sema4A KO recipient mice receiving Th17-skewed WT myelin oligodendrocyte glycoprotein (MOG)-specific encephalitogenic T cells showed a significant reduction in the clinical score compared to the WT recipient mice. However, Sema4A KO recipient mice showed similar disease activity to the WT recipient mice when transferred with Th1-skewed encephalitogenic T cells. Bone marrow chimeric study indicated that Sema4A expressed on hematopoietic cells, but not the CNS resident cells, are responsible for augmenting Th17-mediated neuroinflammation. Additionally, in contrast to comparable IFN-γ levels, IL-17A is significantly higher in RRMS patients with high Sema4A level than those with low Sema4A patients with high Sema4A levels showed earlier disease onset, more severe disease activity and IFN-β unresponsiveness than those with low Sema4A levels.

**Conclusions:**

Sema4A is involved not only in the Th cell priming but also in the acceleration of Th17 cell-mediated neuroinflammation in the effector phase, which could contribute to the higher disease activity observed in RRMS patients with high serum Sema4A levels.

## Background

Semaphorins were originally identified as axon guidance molecules during neural development [1]. However, semaphorins are currently known to have diverse and important functions in other physiological processes, including heart morphogenesis, vascular growth [2], tumor progression [3, 4], and immune cell regulation [5]. Some semaphorins are crucially involved in immune responses, including helper T cell (Th) activation and differentiation [5].

Sema4A is a membrane-type class IV semaphorin that we originally identified as a T cell regulator [6]. Recent studies suggest that Sema4A plays critical roles in many processes including immune cell activation, differentiation, and migration [7]. In other studies, Sema4A is also associated with carcinogenesis and retinal systems [8, 9].

Reflecting the function as T cell activator, the development of myelin oligodendrocyte glycoprotein (MOG)-induced experimental autoimmune encephalomyelitis (EAE), an animal model of multiple sclerosis (MS), is improved in Sema4A-deficient mice partly due to impaired Th1 and Th17 differentiation [6, 10]. Moreover, administration of recombinant Sema4A protein concurrently with IFN-β diminished the efficacy of IFN-β in EAE by promoting encephalitogenic Th1 and Th17 cell differentiation [11]. Consistently, MS patients with high Sema4A levels (≥ 2500 U/ml) exhibit Th17 skewing in peripheral blood mononuclear cells (PBMC) and show significantly more severe Expanded Disability Status Scale (EDSS) score under IFN-β treatment, while fingolimod is effective for those patients [12]. These effects of Sema4A were attributed to the promotion of Th1 and Th17 differentiation in the priming phase and account for non-responsiveness to IFN-β in MS patients with high Sema4A levels. However, the role of Sema4A in the effector phase of neuroinflammation in EAE and MS remains unknown.

Here, we analyzed the association of Sema4A in the effector phase by adoptively transferring either encephalitogenic Th1 or Th17 cells to Sema4A-deficient mice and found that Sema4A play an important role in augmenting Th17-mediated, but not Th1-mediated, neuroinflammation in the effector phase. Consistently, MS patients with high levels of Sema4A showed younger onset, higher disease activity, and higher levels of serum IL-17A.

## Materials and methods

### Patients

Serum Sema4A levels were analyzed in 201 relapsing-remitting multiple sclerosis (RRMS) patients who met the MacDonald criteria 2010 [13] and whose onset age was over 12 years old. Patients with primary progressive MS, clinically isolated syndrome (CIS), and neuromyelitis optica (NMO) or NMO spectrum disorders (NMOSD) were excluded from the study. This study was approved by Osaka University Hospital and other 57 hospitals in Japan. All patients provided informed consent before enrollment in this study. Blood samples were obtained from RRMS patients during the remitting phase. The blood samples were allowed to clot at room temperature, and then the sera were separated by centrifugation and stored at − 80 °C until further use. Cytokine assays were analyzed in 86 RRMS patients at the time of remitting phase. Cytokine assays and no evidence of disease activity (NEDA) assessment were analyzed in RRMS patients (*n* = 86, *n* = 38, respectively) who have been on IFN-β therapy for at least 6 months. These analyses were performed in RRMS patients with high Sema4A levels (≥ 2500 U/ml) and RRMS patients with low Sema4A levels (< 2500 U/ml), in accordance with previous studies [10, 12].

### Outcome measures and procedures

A relapse was defined as the occurrence of new symptoms or exacerbation of existing symptoms persisting for at least 24 h in the absence of concurrent illness or fever (< 37.5 °C) and occurring at least 30 days after a previous relapse.

The annualized relapse rate (ARR) was calculated based on the number of confirmed relapses and total number of the days. ARR was evaluated at the time of Sema4A analysis and IFN-β treatment. Disability scale was evaluated using changes in EDSS. EDSS change was calculated based on the EDSS score and total number of days (1) from onset to analysis of Sema4A or (2) from onset to start of IFN-β treatment. MRI measures included the number of new/newly enlarged T2 lesions and gadolinium-enhanced (Gd+) T1 lesion count. NEDA was defined as a composite that consisted of the absence of clinical relapses, no EDSS score progression (which was defined as either ≥ 1.5-point increase in the EDSS score if baseline EDSS is 0 or ≥ 1 point increase in the EDSS score if baseline EDSS is ≥ 1), and no new or enlarging T2 or T1 Gd+ brain lesions on annual MRI. NEDA was evaluated before and after IFN-β treatment (0, 1, 2, 4, 5 years from the start of IFN-β).

### Sema4A ELISA

Monoclonal antibodies against Sema4A, which recognize both human and mouse Sema4A, were generated as previously described [14]. ELISA plates were coated with the monoclonal anti-Sema4A Abs (Sema4A ELISA kit; MBL). The patient sera were diluted (1:3), and a biotinylated monoclonal anti-Sema4A Ab 5E3 was used as detection Ab. One nanogram per milliliter of recombinant Sema4A-Fc protein, which was used as a standard, was equivalent to 1 U/ml of serum Sema4A.

### Cytokine assay

Sera from RRMS patients were collected, and cytokine profiles (IFN-γ, IL-17, and IL-4) were analyzed using BD™ Cytometric Bead Array assays (BD-bioscience, San Jose, CA, USA).

### Isolation of CD4+ T cell and quantitative PCR

PBMC were collected from RRMS patients with high Sema4A levels (≥ 2500 U/ml, *n* = 14), RRMS patients with low Sema4A levels (< 2500 U/ml, *n* = 12), and health controls (HC) (*n* = 13) by Ficoll-Isopaque density gradient centrifugation (GE Healthcare Japan, Tokyo, Japan). CD4+ T cells were positively separated using an auto MACS cell purification system. RNA of CD4+ T cells was purified using ISOGEN (Nippon Gene Co., Tokyo, Japan) according to the manufacturer’s recommendations. cDNA was generated from a 100-ng RNA sample with an oligo dT primer and ReverTra Ace reverse transcriptase (Toyobo, Osaka, Japan) according to the manufacturer’s recommendations.

Real-time RT-PCR was performed using the thermal cycler TaqMan 7900HT Fast Real-Time PCR System (Applied Biosystems, Carlsbad, CA, USA).

Combined primers and probes were purchased from Applied Biosystems. PCR reactions were performed in triplicate in 20 μl using the TaqMan Universal PCR Master Mix (Applied Biosystems) and 1 μl cDNA. RT-PCR was carried out at 95 °C (10 min), followed by 40 cycles of 94 °C (1 min), 56 °C (1 min), and 72 °C (2 min). The relative expression levels of each mRNA were calculated using the ΔΔCt method and normalized to β-actin. Results are expressed relative to the control group.

### Animals and reagents

Wild type C57BL/6 female mice were purchased from Oriental Yeast Corp. (Tokyo, Japan) and were maintained in a specific pathogen-free environment. Experimental procedures were approved by the Animal Care and Use Committee of Osaka University Graduate School of Medicine (permit number 20-084-6). All possible efforts were made to minimize animal suffering and limit the number of animals used.

### Induction of EAE

EAE was induced using a modification of our previously reported method [15]. In brief, 8-week-old wild type or Sema4A-deficient C57BL/6 female mice were subcutaneously injected with 100 μg MOG_35–55_ emulsified in complete Freund’s adjuvant (CFA) supplemented with intraperitoneal injections of 200 ng pertussis toxin (List Laboratories, Campbell, CA, USA) on days 0 and 2. All mice were monitored daily for clinical signs and were scored as follows using a scale of 0–5: 0, no overt signs of disease; 1, limp tail; 2, hind limb paralysis; 3, complete hind limb paralysis; 4, complete forelimb paralysis; and 5, moribund state or death.

### Adoptive transfer EAE model and IFN-β treatment

For adoptive transfer, donor mice were immunized with MOG/CFA in the same fashion as actively induced EAE in the absence of pertussis toxin. Ten days later, spleens and draining lymph nodes were collected, and single-cell suspensions were prepared. Cells (5 × 10^6^ cells/ml) were cultured with 40 μg/ml MOG_35–55_ peptide and 10 ng/ml recombinant mouse IL-23 (R&D Systems, Minneapolis, MN) in the presence of anti-IL-4 (3 μg/ml) and IFN-γ (3 μg/ml). Three days later, cells were harvested, and CD4+ T cells were isolated by negative selection using Dynabeads (Invitrogen, Carlsbad, CA, USA). Recipient mice irradiated sublethally (500 cGy) received harvested CD4+ T cells intravenously. We assessed the frequency of donor CD4+ T cells producing IL-17 and IFN-γ by FACS analysis before transfer. For IFN-β treatment, IFN-β1b (10,000 U) or phosphate-buffered saline (PBS) was intraperitoneally injected to transferred mice every other day from days 0 to 10 after transfer.

### Establishment of bone marrow chimeric mice

Bone marrow (BM) cells were isolated by flushing femur and tibia bones with Hank’s balanced salt solution (HBSS). BM was filtered through a 100-μm cell strainer, and cells were washed with HBSS. CD45.2 recipient mice were lethally irradiated with 950 cGy and injected i.v. with 2 × 10^6^ CD45.1 BM cells. Engraftment took place over 6–8 weeks of recovery. Mice were bled retro-orbitally to ensure 95% engraftment of blood leukocytes.

### Statistical analyses

**Fig. 1:**
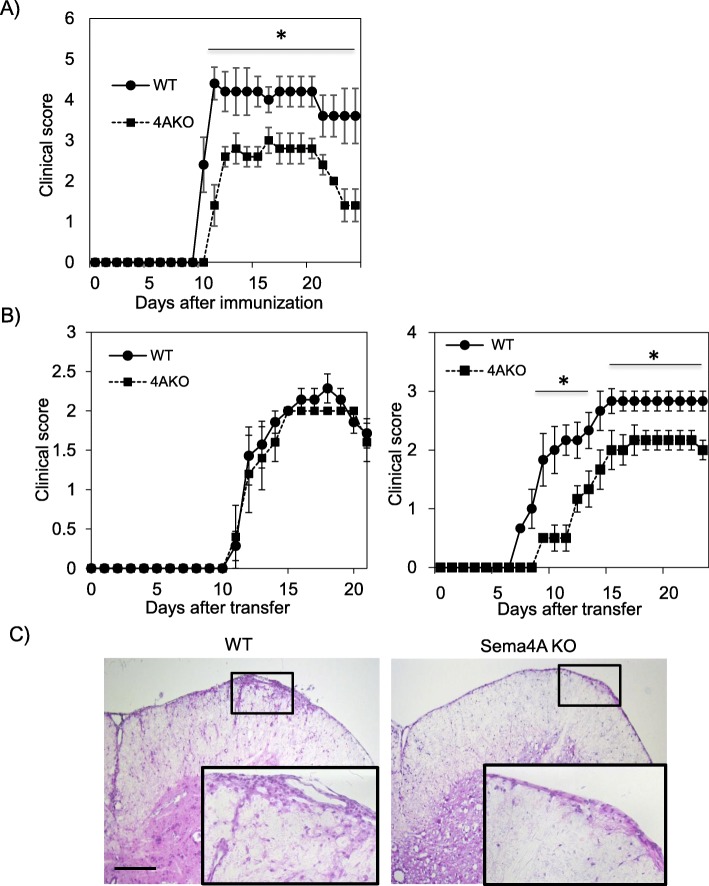
Adoptively transferred Th17, but not Th1, encephalitogenic cells exacerbate EAE in Sema4A-deficient mice. After active immunization, Sema4A KO (4AKO) mice exhibited less severe EAE clinical course than wild type (WT) mice (**a**). Either Th1- or Th17-skewed MOG-specific T cells were adoptively transferred to either WT or Sema4A KO mice (**b**). No significant difference was observed among the mice transferred with Th1-skewed WT MOG-specific encephalitogenic T cells (**b**, left panel). Sema4A KO mice receiving Th17-skewed WT MOG-specific encephalitogenic T cells showed a significant reduction in the clinical score (**b**, right panel). Infiltration of mononuclear cells in the spinal cord of Sema4A KO recipient mice was markedly attenuated when Th17-skewed WT MOG-specific encephalitogenic T cells were transferred (**c**). Data are expressed as means ± SEM. *P < 0.05. Scale bars, 300 μm

**Fig. 2:**
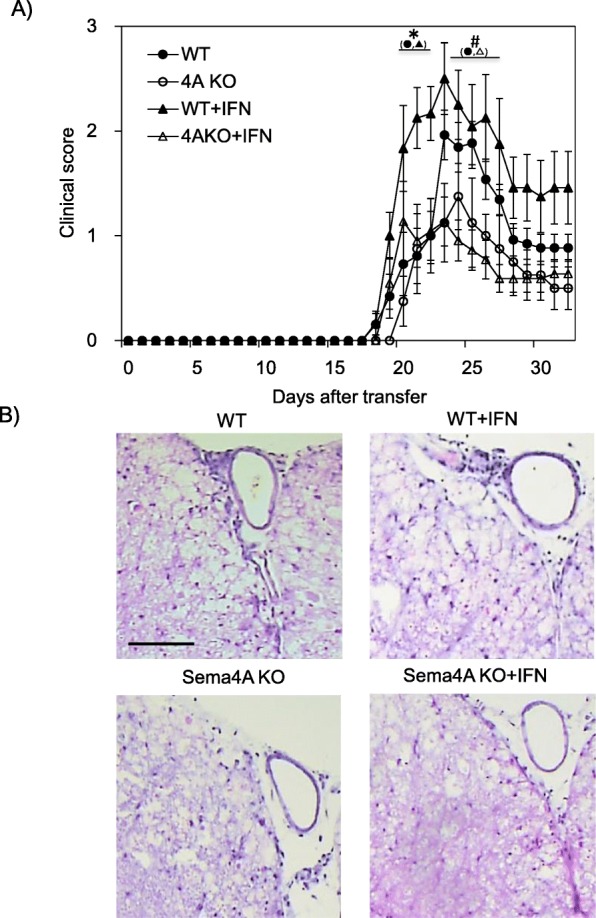
Sema4A KO EAE receiving Th17-skewed encephalitogenic T cells are resistant to IFN-β mediated exacerbation. IFN-β treatment exacerbated clinical score of Th17-induced WT recipient mice; however, no significant change was observed among the Sema4A KO recipient mice (**a**). Infiltration of mononuclear cells was not augmented in the spinal cord of Sema4A KO recipient mice receiving IFN-β treatment (**b**). Data are expressed as means ± SEM. *P < 0.05 for WT vs. WT+IFN; ^#^*P* < 0.05 for WT vs. 4AKO+IFN. Scale bars, 20 μm

**Fig. 3:**
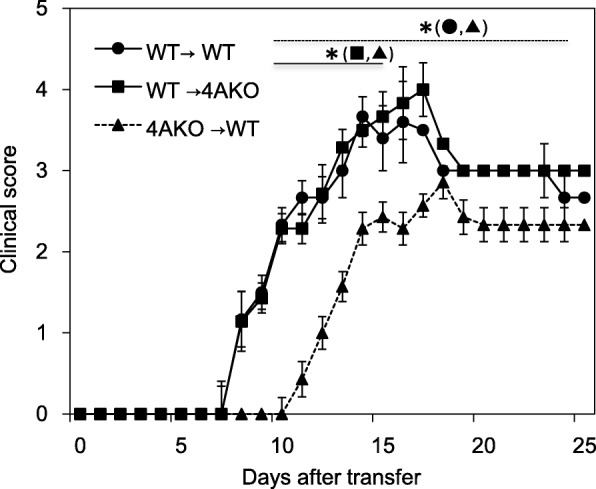
Bone marrow chimera mice transplanted with Sema4A KO cells exhibit amelioration of EAE disease activity. BM chimera mice were generated by transplanting WT or Sema4A KO CD45.1 BM cells to CD45.2 WT or Sema4A KO mice (WT → WT, WT → Sema4A KO, Sema4A KO → WT), and adoptively transferred with WT MOG-specific Th17 cells. WT → Sema4A KO recipient mice showed comparable clinical score to WT → WT recipient mice. By contrast, Sema4A KO → WT recipient mice showed amelioration of EAE clinical score. Data are expressed as means ± SEM. *P < 0.05

**Fig. 4:**
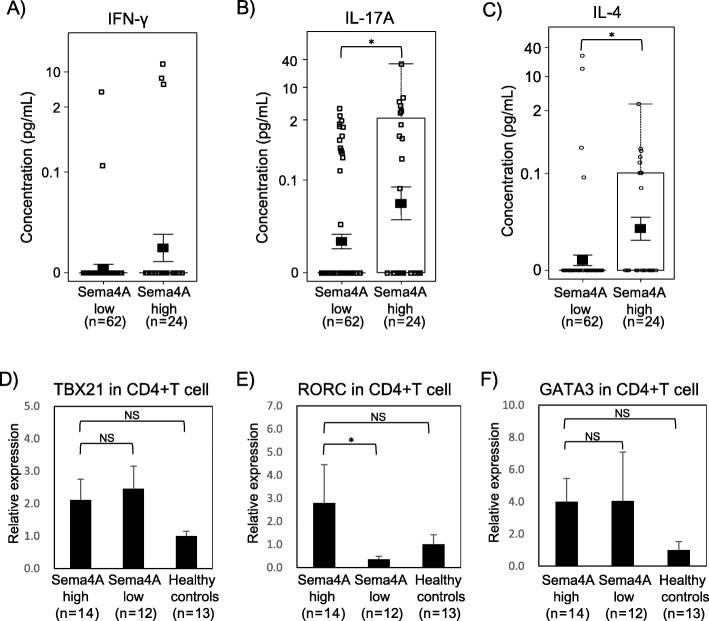
RRMS patients with high Sema4A levels present Th17 but not Th1 immune signature. No significant difference was observed in serum IFN-γ levels between RRMS patients with high (≥ 2500 U/ml) and low Sema4A levels (< 2500 U/ml) (**a**). IL-17A and IL-4 were significantly higher in RRMS patients with high Sema4A level (**b**, **c**). Expression of RORC, but not TBX21 or GATA3, was elevated in CD4+ T cell isolated from PBMC of RRMS patients with high Sema4A levels (**d**–**f**). Data are expressed as means ± SEM. *P < 0.05; NS, not significant (*P* ≥ 0.05)

**Fig. 5:**
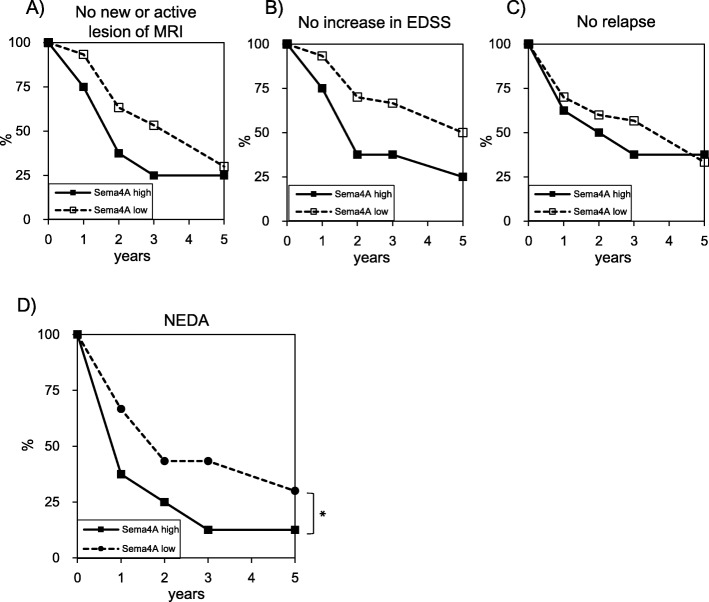
RRMS patients with high Sema4A levels show more severe disease activity. The cumulative disease-free ratios (**a** no new or active lesion of MRI, **b** no increase in EDSS, **c** no relapse, **d** NEDA) after the initiation of INF-β treatment are shown. Years after the initiation of IFN-β treatment are indicated on the *X* axis. 32.1% of patients with low Sema4A significantly achieved NEDA at 5 years, while 12.5% of patients with high Sema4A maintained NEDA status 5 years after the start of IFN-β treatment (**d**). *P < 0.05

Continuous variables were expressed as the mean ± SE. Student’s *t* test was usually used to compare continuous variables between two independent groups when the data tend to follow normal distribution, while Welch’s *t* test was selected if the SDs of the two groups were highly different or Mann-Whitney *U* test was selected if the data do not tend to follow normal distribution. For categorical variables, the groups were compared using Pearson’s chi-squared test for categorical outcomes. All reported *p* values are two-sided, and *p* values < 0.05 were considered statistically significant and were marked by an asterisk (*) in Table 1 and Figs. 1, 2, 3, and 4. Statistical analysis in Fig. 5 was performed using the logistic mixed model to compare repeated binary outcomes between two groups because each achievement item on the same patients is repeatedly evaluated over 5 years. These statistical analyses were conducted using SPSS 16.0 J (SPSS Japan Inc., Tokyo, Japan) and the R programming language.

**Table 1:** Clinical characteristics of the patients

	Sema4A high (≥ 2500 U/ml) (*n* = 63)	Sema4A low (< 2500 U/ml) (*n* = 138)	*P*
Female/male (% female)	51/12 (81.0)	112/26 (81.2)	0.972
Age at onset (mean ± SD, years)	28.8 ± 8.4	34.8 ± 9.7	0.00004*
Disease duration from onset to examination (mean ± SD, years)	6.3 ± 8.5	8.1 ± 6.9	0.125
Sema4A (mean ± SD, U/mL)	26,003.7 ± 108,879.5	439.9 ± 635.2	–
Positive ratio of OCB (*n*/*N*, (%))	29/46 (63.0)	54/104 (51.9)	0.207
Distribution of MRI lesions (*n*/*N*, (%))			
- Cerebrum	59/62 (95.2)	129/138 (93.5)	0.643
- Cerebellum	10/60 (16.7)	25/138 (18.1)	0.806
- Brain stem	27/62 (43.5)	72/138 (52.2)	0.259
- Spinal cord	45/61 (73.8)	93/137 (67.9)	0.405
- Optic nerve	22/61 (36.1)	45/136 (33.1)	0.683
EDSS change before Sema4A analysis (mean ± SD, /year)	0.7 ± 0.5	0.4 ± 0.5	0.01*
ARR before Sema4A analysis (mean ± SD, /year)	1.13 ± 0.94	0.83 ± 0.74	0.04*

## Results

### Adoptively transferred Th17, but not Th1, encephalitogenic cells exacerbate EAE in Sema4A-deficient mice

To confirm the relevance of Sema4A in EAE development, actively immunized EAE was induced in either wild type (WT) or Sema4A-deficient (Sema4A KO) mice. Consistent with the previous report, Sema4A KO mice exhibited less severe EAE clinical course (Fig. 1a). To perform more detailed analysis of how Sema4A is related to neuroinflammation at the effector phase, either Th1- or Th17-skewed MOG-specific T cells were adoptively transferred to either WT or Sema4A KO mice (Fig. 1b). When Th1-skewed WT MOG-specific encephalitogenic T cells were transferred to WT or Sema4A KO recipient mice, no significant difference was observed between the two groups. By contrast, Sema4A KO mice receiving Th17-skewed WT MOG-specific encephalitogenic T cells showed a significant reduction in the clinical score compared to the WT recipient mice (Fig. 1b). Consistent with the clinical results, infiltration of mononuclear cells in the spinal cord of Sema4A KO recipient mice were markedly attenuated when Th17-skewed WT MOG-specific encephalitogenic T cells were transferred (Fig. 1c), suggesting that Sema4A serves as a disease-augmenting factor at the effector phase specifically in Th17-, but not Th1-, induced inflammatory condition with the CNS.

### Th17-induced Sema4A KO EAE were resistant to IFN-β-mediated exacerbation

The previous report showed that IFN-β treatment exacerbated Th17-mediated EAE. To determine the relevance of Sema4A on Th17-mediated deterioration of EAE under IFN-β treatment, we adoptively transferred WT MOG-specific Th17 cells to WT or Sema4A-KO recipient mice and then treated both mice with IFN-β. As previously reported, IFN-β treatment exacerbated clinical score of Th17-induced WT recipient mice (Fig. 2a). Of note, IFN-β treatment did not change the clinical course of Th17-induced Sema4A KO recipient mice (Fig. 2a). Histological analysis also confirmed that infiltration of mononuclear cells was not augmented in the spinal cord of Sema4A KO recipient mice receiving IFN-β treatment (Fig. 2b). These results suggest that Sema4A plays a critical role in IFN-β-induced augmentation of Th17-mediated neuroinflammation.

### Sema4A expressed on hematopoietic cells determines augmentation of Th17 cell-mediated EAE exacerbation

To clarify whether Sema4A expressed on resident CNS cells are contributing to the augmentation of Th17-mediated neuroinflammation, we generated BM chimera mice by transplanting WT or Sema4A KO CD45.1 BM cells to CD45.2 WT or Sema4A KO mice (WT → WT, WT → Sema4A KO, Sema4A KO → WT), and adoptively transferred WT MOG-specific Th17 cells to these chimeric mice. While WT → Sema4A KO recipient mice showed comparable clinical score to WT → WT recipient mice, Sema4A KO → WT recipient mice showed amelioration of clinical score and delayed disease onset (Fig. 3). These results indicate that Sema4A expressed on hematopoietic cells, but not on the CNS resident cells, are responsible for augmenting Th17-mediated neuroinflammation.

### RRMS patients with high Sema4A levels present Th17 but not Th1 immune signature

Th1 and Th17 cells are main effector Th subsets in the autoimmune and inflammatory process of MS [16]. To examine the main effector subset in MS with high serum Sema4A levels, we compared serum levels of IFN-γ and IL-17A among RRMS patients with high (≥ 2500 U/ml) and low Sema4A levels (< 2500 U/ml). Although no significant difference was observed in IFN-γ levels between the two groups (Fig. 4a), IL-17A was significantly higher in RRMS patients with high Sema4A levels (*p* = 0.0414) (Fig. 4b). In addition, quantitative PCR analysis revealed that expression of RORC, but not TBX21, was elevated in CD4+ T cell isolated from PBMC of RRMS patients with high Sema4A levels (Fig. 4d, e). Recently, Lu et al. reported that human Sema4A regulate Th2 differentiation [17]. We analyzed IL-4 level in the sera and GATA3 expression in CD4+ T cells in RRMS patients. Il-4 was increased in RRMS with high Sema4A, although no significant difference was observed in the expression of GATA3 between patients with high Sema4A levels and patients with low Sema4A levels (Fig. 4c, f). These data suggest that Th17 rather than Th1 cells are the main effector Th cells involved in the pathogenesis of RRMS patients with high Sema4A levels.

### RRMS patients with high Sema4A levels show earlier onset and more severe disease activity

To clarify the disease activity of patients with high Sema4A levels, detailed clinical and radiological analysis was performed. Patients with high Sema4A levels showed a significantly earlier age of onset (28.8 ± 8.4 versus 34.8 ± 9.7) (*p* = 0.00004), higher EDSS change (0.7 ± 0.5 versus 0.4 ± 0.5) (*p* = 0.01), and higher ARR (1.13 ± 0.94 versus 0.83 ± 0.74) (*p* = 0.04, Table 1). No significant differences were found in the positive ratio of oligoclonal immunoglobulin G bands (63% versus 51.9%), distribution of MRI lesions, and EDSS score at the time of Sema4A analysis (Table 1). Nine of 28 (32.1%) patients with low Sema4A achieved NEDA at 5 years, while only 1 of 8 (12.5%) patients with high Sema4A maintained NEDA status after 5 years (*p* = 0044). The ratios of disease-free patients for three individual measures (relapse, EDSS score, MRI) in RRMS patients with high Sema4A levels tended to be lower (33.3% versus 37.5%, 50.0% versus 25%, and 30.0% versus 25.0%) than those with low Sema4A levels (Fig. 5). These data suggest that Sema4A is associated with the disease onset and the disease activity of RRMS.

## Discussion

We identified that Sema4A promotes Th17-induced neuroinflammation in the effector phase using EAE induced by adoptive transfer of Th17 cells. In the context of EAE and MS, marked infiltration of monocytes following lymphocyte recruitment is recognized to be a critical factor forming the destructive lesions in the CNS [18]. Sema4A is expressed on monocyte in the human blood [10]. In addition, infiltrating macrophages in MS brain abundantly express Sema4A [19]. Consistent with these findings, the lack of Sema4A expression on hematopoietic cells caused a less severe disease course and delayed onset, compared with chimeric mice that express Sema4A in hematopoietic cells or that lack Sema4A in CNS resident cells. Our observations suggest that Sema4A on monocytes play an essential role in augmenting the tissue inflammation in CNS autoimmunity. A recent study reported that hematopoietic stem cell transplantation can be more efficacious for patients with RRMS compared with currently well-acknowledged disease modifying therapies [20]. MS patients who received autologous hematopoietic stem cell transplantation reportedly show recovery of monocyte expansion by 6 months after transplantation [21]. Our findings indicate some possibility that patients with high Sema4A levels might be susceptible to higher risk of relapses if the monocyte number and the levels of Sema4A return to the same level as the baseline after the application of transplantation.

IFN-β inhibits Th1 and Th17 differentiation, while Sema4A abrogate these processes. However, it remains unclear whether IFN-β affects Sema4A function in the effector phase, Axtell et al. reported that IFN-β exacerbates Th17-induced EAE [22], while it improves Th1 induced EAE. In this study, we confirmed that IFN-β exacerbated Th17-induced EAE in WT recipients, but not in Sema4A KO recipients. These data together suggest that Sema4A associates with IFN-β resistance not only by inhibiting therapeutic effect in Th priming but also by augmenting Th17 immunity in the effector phase. Originally, Sema4A was identified as an activator of Th cell. In a murine model, Sema4A is also reported to promote Th1 differentiation. Regarding RRMS patients, intracellular cytokine staining of PBMC previously revealed Th17 skewing in RRMS patients with high Sema4A levels [10]. In this study, we confirmed a significant increase of serum IL-17A in the remission period of RRMS, while IFN-γ was not changed. Il-4 was also increased in RRMS with high Sema4A, although no significant difference was observed in the expression of GATA3 between patients with high Sema4A levels and patients with low Sema4A levels. Th2 immunity plays a protective role in MS pathogenesis [23]. However, RRMS patients with high Sema4A levels showed higher disease activity. Therefore, it was indicated that Th17 cells have a more pivotal role than Th2 cells in patients with high Sema4A levels. Collectively, our findings suggest that RRMS patients with high Sema4A levels are more susceptible to Th17 skewed peripheral immunity in the steady state and that Th17 cells are main effector Th cell in the pathogenesis of RRMS patients with high Sema4A levels.

Interestingly, RRMS patients with high Sema4A show younger disease onset and have a high level of Sema4A at the stage of CIS, which is not changed by treatment or relapses [10]. In this study, patients with high Sema4A levels also showed higher EDSS change and ARR, indicating more augmented disease activity. Recently to define the treatment efficacy of MS therapies, the concept of NEDA is often applied in clinical trials [24]. Considering the fact that NEDA encompass three domains of relapses, MRI activity, and EDSS, NEDA can also be regarded as a combined scale reflecting the inflammatory activity within the CNS. As shown in our study, patients with high Sema4A levels are significantly less likely to maintain NEDA after 5 years. Thus, in addition to pro-inflammatory nature of Sema4A observed in EAE, clinical observation also highlights that patients with high Sema4A levels are more prone to show clinical course of higher disease activity.

Taken together, these observations support the possibility that Sema4A may facilitate disease onset of RRMS by promoting Th17 differentiation during the process of antigen-specific Th cell priming and furthermore may accelerate Th17 cell-mediated neuroinflammation in the effector phase, resulting in higher disease activity of patients with high Sema4A levels.

## Conclusions

This study demonstrated that Sema4A is involved not only in the Th cell priming but also in the acceleration of Th17 cell-mediated neuroinflammation in the effector phase, which could contribute to the higher disease activity observed in RRMS patients with high serum Sema4A levels.

## Data Availability

The datasets generated during and/or analyzed during the current study are available from the corresponding author upon reasonable request.

## Abbreviations

ARR: Annualized relapse rate
BM: Bone marrow
CFA: Complete Freund’s adjuvant
CIS: Clinically isolated syndrome
CNS: Central nervous system
EAE: Experimental autoimmune encephalomyelitis
EDSS: Expanded Disability Status Scale
MOG: Myelin oligodendrocyte glycoprotein
MS: Multiple sclerosis
NEDA: No evidence of disease activity
NMO: Neuromyelitis optica
NMOSD: NMO spectrum disorders
PBMC: Peripheral blood mononuclear cells
RRMS: Relapsing-remitting multiple sclerosis
Sema4A KO: Sema4A-deficient
Th: Helper T cell
WT: Wild type
